# Cilostazol for heart rate increase in atrial fibrillation with slow ventricular response: an exploratory randomized controlled trial

**DOI:** 10.1093/ehjopen/oeag068

**Published:** 2026-04-27

**Authors:** Gabriela Hinkelmann Berbert, Rogerio Braga Andalaft, Bruno Pereira Valdigem, Luciana Vidal Armaganijan, Fabio Antonio Venancio, Dalmo Antonio Ribeiro Moreira

**Affiliations:** Electrophysiology Department, Instituto Dante Pazzanese de Cardiologia, Sao Paulo, Brazil; Arrhythmia Center, Einstein Hospital Israelita, Sao Paulo, SP, Brazil; Electrophysiology Department, Instituto Dante Pazzanese de Cardiologia, Sao Paulo, Brazil; Arrhythmia Center, Einstein Hospital Israelita, Sao Paulo, SP, Brazil; Electrophysiology Department, Instituto Dante Pazzanese de Cardiologia, Sao Paulo, Brazil; Arrhythmia Center, Einstein Hospital Israelita, Sao Paulo, SP, Brazil; Electrophysiology Department, Instituto Dante Pazzanese de Cardiologia, Sao Paulo, Brazil; Electrophysiology Department, Instituto Dante Pazzanese de Cardiologia, Sao Paulo, Brazil; Nursing Department, Centro Universitário de Adamantina, Sao Paulo, SP, Brazil; Electrophysiology Department, Instituto Dante Pazzanese de Cardiologia, Sao Paulo, Brazil

**Keywords:** Atrial Fibrillation, Bradycardia, Cilostazol, Pacemaker Alternatives

## Abstract

**Aims:**

Atrial fibrillation (AF) with slow ventricular response is a clinically challenging condition, particularly in elderly patients with multiple comorbidities. Current therapeutic options for the chronic pharmacological management of bradycardia remain limited. The aim of this study was to evaluate the effects of cilostazol on heart rate (HR) and ventricular arrhythmia in patients with permanent AF and a slow ventricular response.

**Methods and results:**

This was a randomized, double-blind, placebo-controlled trial including 26 patients with permanent AF and a mean HR <65bpm on 24-hour Holter monitoring. Patients were randomized to receive cilostazol 100 mg twice daily (*n* = 14) or placebo (*n* = 12) for 3 months. The primary endpoint was the increase in mean HR. After 30 days of treatment, the cilostazol group demonstrated a significant increase in minimum HR (from 34.17 to 39.14 bpm; *P* = 0.032) and mean HR (from 55.42 to 64.43 bpm; *P* = 0.013) compared to placebo. The total number of QRS complexes increased significantly (from 73879 to 85249 *P* = 0.029), and the number of cardiac pauses (>2.5 s) was significantly reduced (mean from 56.25 to 2.57 and median from 5.5 to 0.0 *P* = 0.018). No significant differences were observed in the incidence of ventricular arrhythmias. Adverse effects were mild and included headache and gastrointestinal symptoms, with no statistical difference between groups.

**Conclusion:**

Cilostazol significantly increased HR in patients with AF and slow ventricular response without increasing ventricular arrhythmias. These findings suggest that cilostazol may represent a pharmacological alternative for selected patients when pacemaker implantation is not immediately feasible.

## Graphical Abstract

**Graphical Abstract:**
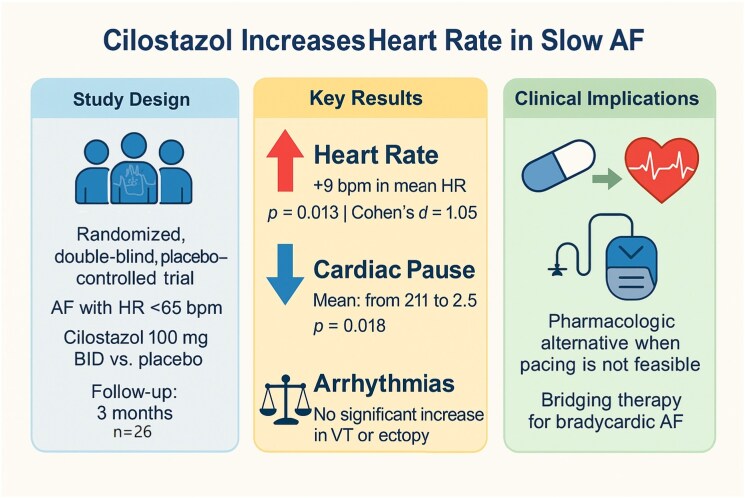

What’s New?

- Randomized, double-blind, placebo-controlled trial to evaluate cilostazol as a pharmacologic alternative to pacing in patients with atrial fibrillation (AF) and slow ventricular response.
- Significant increase in both minimum and mean heart rates and a marked reduction in ventricular pauses on 24-hour Holter monitoring, confirming cilostazol’s positive chronotropic effect in a population traditionally managed with device therapy.
- No increase in ventricular arrhythmias or serious adverse events was observed, supporting cilostazol’s cardiac safety.
- Findings broaden the therapeutic spectrum for bradycardia management in AF, bridging pharmacologic modulation and device-based pacing strategies.

## Introduction

Atrial fibrillation (AF) is the most common sustained arrhythmia, and its prevalence continues to increase.^1^ Although often asymptomatic, AF is associated with major adverse outcomes. Therapeutic management of AF remains challenging, resulting in a significant proportion of patients living chronically with the arrhythmia.^1,2^

Approximately 20% of patients with AF present with sick sinus syndrome (SSS), reflecting shared fibrotic remodelling of atrial and conduction tissues, leading to AF with slow ventricular response. SSS remains the main indication for permanent pacemaker implantation worldwide.^3^ This clinical scenario creates a therapeutic challenge, as pharmacological options to increase HR remain limited.

Cilostazol is a selective and reversible phosphodiesterase type 3 (PDE3) inhibitor. Beyond its well-established antiplatelet and vasodilatory effects, cilostazol has also demonstrated pleiotropic actions. Unlike other PDE3 inhibitors, cilostazol also inhibits cellular adenosine uptake, which may further enhance cAMP levels and contribute to its distinct cardiovascular effects.^4–6^

A meta-analysis including more than 2700 patients confirmed its overall safety profile and consistently reported modest increases in HR (∼7bpm), particularly among bradycardic patients, supporting its potential role as a chronotropic agent.^6^

Currently, there are no formal guideline recommendations for chronic pharmacological therapy in the management of bradycardia, with therapeutic options being limited to permanent pacemaker implantation or clinical monitoring. In such scenarios, particularly in oligosymptomatic patients or when symptom attribution is uncertain, therapeutic options remain limited.

## Methods

This was a randomized, double-blind, placebo-controlled trial conducted at a tertiary cardiology centre. A total of 26 patients with permanent AF and slow ventricular response (mean HR < 65 bpm) were included. Patients were randomized to receive cilostazol (*n* = 14) or placebo (*n* = 12).

Sample size calculation assumed a minimum clinically relevant difference of 10bpm between groups, with a standard deviation of 5bpm, a two-sided α of 0.05, and 95% power. This resulted in a minimum required sample of 14 participants (7 per group).

All participants were evaluated by an independent electrophysiology team, and none had an indication for permanent pacemaker implantation. Participants were excluded if they were taking negative chronotropic agents or had any contraindication to cilostazol use, including heart failure, a known major contraindication to the drug.

**Figure 1:**
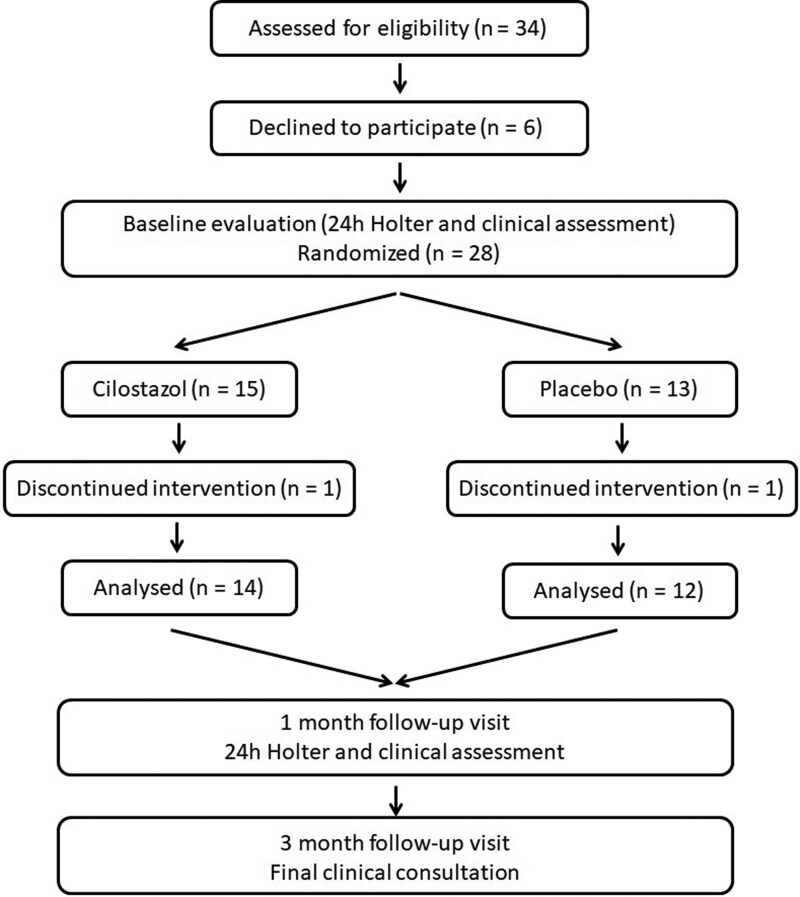
Study flow diagram. Flow diagram summarizing patient screening, randomization, and follow-up in the study. A total of 26 patients with atrial fibrillation and slow ventricular response were randomized to receive cilostazol or placebo and completed the study follow-up.

Patients were randomized in blocks of six to receive cilostazol 100 mg BID or placebo orally. Study medications and matching placebos were sequentially numbered to ensure allocation concealment. The study flow diagram is presented in *Figure 1*. Echocardiographic and laboratory data were obtained from electronic medical records before study inclusion. Because heart failure was an exclusion criterion, all participants had normal LVEF.

## Endpoints

The primary endpoint was the change in mean HR after treatment. Secondary endpoints included treatment safety and the occurrence of ventricular arrhythmias.

## Ethics and consent

The study was conducted in accordance with the principles of the Declaration of Helsinki. The study protocol was approved by the local Research Ethics Committee and by the Brazilian National Research Ethics Commission (CONEP), under Certificate of Presentation for Ethical Consideration (CAAE) number 38699020.0.0000.5462. Written informed consent was obtained from all participants prior to enrolment.

## Statistical analysis

Normality was assessed using the Shapiro-Wilk test. For normally distributed variables, Student’s *t*-test was applied (paired for within-group and independent for between-group comparisons). For non-parametric variables, the Wilcoxon signed-rank test (paired) and the Mann–Whitney *U* test (independent) were used.

Effect size was calculated using Cohen’s d for t-tests and r for Wilcoxon/Mann–Whitney tests. *P* < 0.05 was adopted. Statistical analyses were performed using R software (version 4.4.1).

## Results

The study included 26 participants randomized to receive cilostazol (*n* = 14) or placebo (*n* = 12). Demographic and clinical characteristics were similar, with no statistically significant differences between groups. The population characteristics are summarized at *Table 1*.

**Table 1:** Population characteristics

	Randomization group						
	Cilostazol		Placebo		Total		*P*-value^a^
	*n*	%	*n*	%	*n*	%	
**Gender**							**0**.**254**
Female	6	40.00	8	61.54	14	50.00	
Male	9	60.00	5	38.46	14	50.00	
**Age**							**0**.**652**
< 65 years old	0	0.00	2	15.38	2	7.14	
66–70 years	2	13.33	0	0.00	2	7.14	
71–75 years	3	20.00	3	23.08	6	21.43	
76–80 years	4	26.67	3	23.08	7	25.00	
81–85 years	4	26.67	3	23.08	7	25.00	
86–90 years	1	6.67	1	7.69	2	7.14	
>90 years	1	6.67	1	7.69	2	7.14	
**BMI** ^b^ **class**							**0**.**615**
Eutrophy	6	40.00	3	23.08	9	32.14	
Obesity	4	26.67	5	38.46	9	32.14	
Overweight	5	33.33	5	38.46	10	35.71	
**Hypertension**							**0**.**193**
No	4	26.67	1	7.69	5	17.86	
Yes	11	73.33	12	92.31	23	82.14	
**Prior stroke**							**0**.**754**
No	3	20.00	2	15.38	5	17.86	
Yes	12	80.00	11	84.62	23	82.14	
**AMI** ^c^							**0**.**473**
No	14	93.33	11	84.62	25	89.29	
Yes	1	6.67	2	15.38	3	10.71	
**Diabetes mellitus**							**0.464**
No	9	64.3	6	50.0	15	57.7	
Yes	5	35.7	6	50.0	11	42.3	
**Peripheral arterial disease**							**0.271**
No	14	100.0	11	91.7	25	96.2	
Yes	0	0.0	1	8.3	1	3.8	
**Chronic Kidney disease**							**0.344**
No	13	92.9	12	100.0	25	96.2	
Yes	1	7.1	0	0.0	1	3.8	

^a^Fisher’s test.

^**b**^Body mass index.

^**c**^Acute myocardial infarction.

During the clinical follow-up, blood pressure did not change significantly within or between groups. No patient required pacemaker implantation during the 3-month study period.

**Figure 2:**
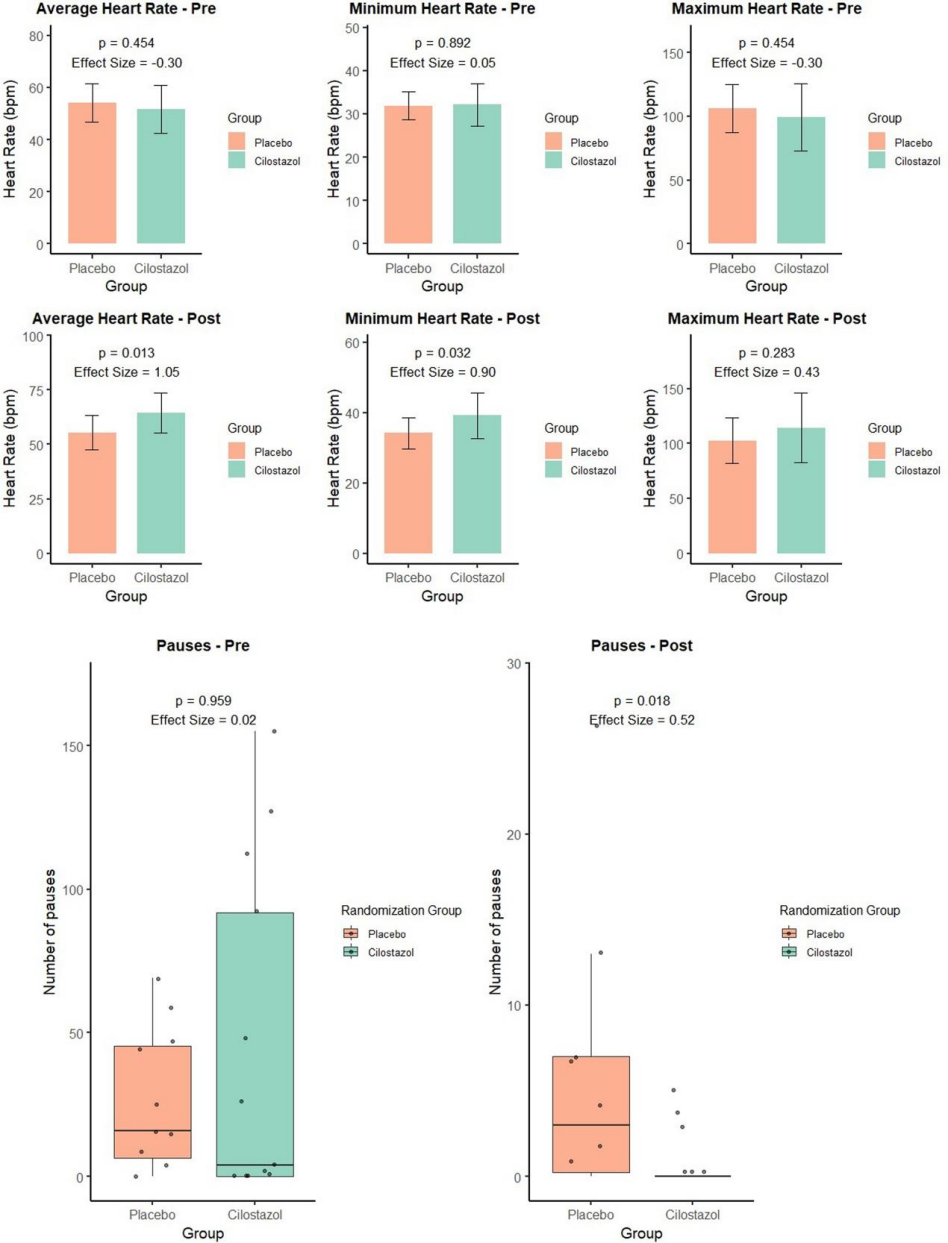
Study results. Boxplots illustrating changes in heart rate and cardiac pauses after 30 days of treatment with cilostazol compared with placebo in patients with atrial fibrillation and slow ventricular response. The cilostazol group showed a significant increase in mean heart rate (∼9 bpm) and a marked reduction in the number of cardiac pauses. bpm = beats per minute; *P* = *P*-value.

After 30 days of treatment, the cilostazol group had significantly higher minimum (39.14 vs. 34.17bpm; *P* = 0.032; d = 0.90) and mean HR (64.43 vs. 55.42bpm; *P* = 0.013; d = 1.05) compared to placebo. Maximum HR did not differ significantly between groups post-intervention (114.36 vs. 102.67bpm; *P* = 0.283). These results are presented in *Figure 2*.

The total number of QRS complexes significantly increased in the cilostazol group compared to placebo (85.249 vs. 73.879; *P* = 0.029; d = 0.91), while the median number of cardiac pauses significantly decreased (median 5.5 vs. 0.0; *P* = 0.018; d = 0.52).

The incidence of ventricular tachycardia or accelerated ventricular rhythm was similar between groups, with no significant differences observed pre- (36% vs. 50%; *P* = 0.464) and post-intervention (57% vs. 58%; *P* = 0.951). Additionally, there were no significant differences in ventricular ectopic beats.

Adverse effects were mild, reported by 42.9% of cilostazol users compared to 25% in the placebo group (*P* = 0.343), predominantly headache and gastrointestinal symptoms, with no patients requiring discontinuation.

## Discussion

This study demonstrates that cilostazol significantly increases HR in patients with AF and slow ventricular response, while maintaining a favourable safety profile. In a predominantly elderly population with multiple comorbidities, treatment with cilostazol was associated with higher minimum and mean HR, a reduction in cardiac pauses, and no increase in ventricular arrhythmias. Not only was the *P* value statistically significant, but the observed effect sizes (Cohen’s d analysis) were also large, indicating a robust therapeutic effect despite the limited sample size. The comparable baseline characteristics between groups further support the internal validity of these findings.

Previous observational studies and small series in patients with sinus node dysfunction or AF with slow ventricular response have reported similar chronotropic effects with cilostazol.^7^ However, most of these studies were non-randomized. Our findings extend this evidence by demonstrating the chronotropic effect of cilostazol within a randomized, double-blind, placebo-controlled framework.

The observed increase in HR is likely related to cilostazol’s inhibition of PDE3, leading to increased intracellular cAMP levels and enhanced calcium channel activity within nodal tissue. In addition, cilostazol partially inhibits adenosine uptake, which may further contribute to its chronotropic properties. Together, these mechanisms may facilitate atrioventricular conduction without producing substantial inotropic stimulation or arrhythmogenic effects. Nevertheless, given the small sample size and short follow-up of this study, these findings should be considered hypothesis-generating.

Importantly, cilostazol did not increase the incidence of ventricular arrhythmias in this study. Given the complexity of the population in the tertiary hospital where the study was conducted, it is noteworthy that the prevalence of ventricular arrhythmias was relatively high in both groups, yet their occurrence remained similar between groups. Adverse events were generally mild and consistent with the known tolerability profile of the drug.

From a clinical perspective, cilostazol may represent a pharmacological alternative or bridging strategy in patients with AF and bradycardia who are not immediate candidates for permanent pacemaker implantation. This may include patients with temporary contraindications to device therapy, diagnostic uncertainty regarding symptom attribution, or those who decline pacing. The observed reduction in cardiac pauses further supports the potential haemodynamic relevance of this therapeutic approach.

This study has several limitations. The relatively small sample size and the short follow-up period limit the ability to assess long-term outcomes and rare adverse events. In addition, the study population consisted predominantly of elderly patients with multiple comorbidities, which may restrict generalizability. Nevertheless, the randomized design and the magnitude of the observed effect (Cohen’s d = 1.05 for mean HR increase) provide meaningful preliminary evidence supporting the chronotropic efficacy of cilostazol in this clinical setting.

Larger studies with longer follow-up are warranted to confirm these findings, evaluate long-term clinical outcomes, and better define the role of cilostazol in the management of bradycardic AF. Nonetheless, this study contributes valuable clinical evidence supporting cilostazol’s utility in specific patient subsets with AF and slow ventricular response.

## Conclusion

Bradycardia in patients with AF may lead to significant haemodynamic impairment and often represents an indication for permanent pacemaker implantation. However, device therapy may not always be immediately available or feasible in clinical practice.

In this randomized, double-blind trial, cilostazol significantly increased HR and reduced ventricular pauses in patients with AF and slow ventricular response without increasing ventricular arrhythmias or serious adverse events. These findings suggest that cilostazol may represent a pharmacological alternative or bridging strategy in selected patients with bradycardic AF.

Larger studies with longer follow-up are warranted to confirm these results and further define the role of cilostazol in this clinical setting.

## Data Availability

The data underlying this article will be shared on reasonable request to the corresponding author.
